# Ultra-processed foods consumption and health-related outcomes: a systematic review of randomized controlled trials

**DOI:** 10.3389/fnut.2024.1421728

**Published:** 2024-06-26

**Authors:** Adolfo Aramburu, Giancarlo Alvarado-Gamarra, Rubelio Cornejo, Katherine Curi-Quinto, Carmen del Pilar Díaz-Parra, Gabriela Rojas-Limache, Claudio F. Lanata

**Affiliations:** ^1^Instituto de Investigación Nutricional, Lima, Peru; ^2^Faculty of Science Health, Universidad Peruana de Ciencias Aplicadas, Lima, Peru; ^3^Department of Pediatrics, Hospital Nacional Edgardo Rebagliati Martins, Lima, Peru; ^4^Department of Pediatrics, School of Medicine, Vanderbilt University, Nashville, TN, United States

**Keywords:** processed food, ultra-processed foods, non-communicable diseases, food consumption, diet, systematic review

## Abstract

**Introduction:**

The increase in ultra-processed foods (UPFs) intake has raised concerns about its impact on public health. Prospective observational studies have reported significant associations between higher intake of UPFs and adverse health outcomes. The aim of this study is to determine whether these associations could be confirmed in randomized controlled trials (RCTs).

**Methods:**

We conducted a systematic review to analyze the evidence on the effects of UPFs intake on health. A systematic search was conducted in Medline, Embase, Web of Science, Scopus, LILACS, and CENTRAL up to April 22, 2024. RCTs in English, Spanish, and Portuguese evaluating the health effects of interventions to modify UPFs intake were included. The certainty of evidence was determined using the GRADE methodology.

**Results:**

Three educational intervention studies and one controlled feeding trial were included, evaluating the effect of reducing the consumption of UPFs (455 participants, median follow-up, 12 weeks). No significant effects were observed in 30 out of the 42 outcomes evaluated. The controlled feeding trial in adults with stable weight showed a reduction in energy intake, carbohydrates, and fat (low certainty of evidence), as well as in body weight, total cholesterol, and HDL cholesterol (moderate certainty of evidence). In the educational intervention studies, a reduction in body weight and waist circumference was observed (low certainty of evidence) in women with obesity, as well as improvement in some dimensions of quality of life (very low certainty of evidence). No significant changes were observed in children and adolescents with obesity, while in overweight pregnant women, the consumption of UPFs was not reduced, so the observed benefits could be attributed to other components of the intervention.

**Conclusion:**

Interventions aimed at reducing the consumption of UPFs showed benefits on some anthropometric and dietary intake outcomes, although significant effects were not observed for most of the evaluated outcomes. The limited number and significant methodological limitations of the studies prevent definitive conclusions. Further well-designed and conducted RCTs are needed to understand the effects of UPF consumption on health.

**Systematic review registration**: https://www.crd.york.ac.uk/prospero/display_record.php?ID=CRD42023469984

## 1 Introduction

Ultra-processed foods (UPFs) are industrial formulations made from modified and unmodified substances extracted from foods, which include few or no whole foods (1). These products result from intensive industrial processing and contain food additives such as preservatives, emulsifiers, flavorings, bulking agents, among others, which are used to extend the product’s shelf life and improve its sensory qualities (2–7). Most UPFs are characterized by high energy density, being rich in saturated fats, refined starches, free sugars, and salt, and low in dietary fiber, proteins, and micronutrients (4, 6).

Worldwide, the consumption of UPFs has experienced a rapid increase, representing more than half of the daily calories consumed in high-income countries (3, 7, 8). Similarly, in low- and middle-income countries, a sustained growth in the sale of these products and their contribution to energy intake is observed (4, 9). The significant increase in UPFs consumption and their dominant role in food systems has sparked extensive debate about their potential impact on public health and has attracted the interest of numerous researchers worldwide (10–12).

Previous systematic reviews have reported an association between high intake of UPFs and all-cause mortality, as well as with specific adverse outcomes such as cardiovascular diseases, metabolic syndrome, type 2 diabetes, overweight, and certain types of cancer (13). However, the evidence included primarily comes from observational studies, which cannot establish causality relationships and may be susceptible to biases and difficulties in controlling potential confounding factors (14). For this reason, we conducted a systematic review with the aim of analyzing the available scientific evidence from randomized controlled trials on the health effects of UPFs consumption, to improve understanding and establish more robust conclusions about these relationships.

## 2 Materials and methods

The present systematic review was conducted following the Preferred Reporting Items for Systematic Reviews and Meta-Analyses (PRISMA) guidelines (see Supplementary Table S1) (15). The study protocol was registered on the PROSPERO platform with the registration number CRD42023469984.

### 2.1 Literature search

A systematic search was conducted in the Medline (PubMed), Embase, Web of Science, Scopus, LILACS, and CENTRAL databases up to April 22, 2024, with no restriction on publication date. The search strategy was developed using a combination of free-text terms and controlled vocabulary (thesaurus) related to UPFs, such as ‘ultra-processed food’, ‘ultra-processed diet’, ‘ultra-processed meal’, and ‘processed food’, as well as terms related to randomized clinical trials (RCTs), including ‘randomized controlled trial’, ‘controlled clinical trial’, ‘RCT’ and ‘crossover’. The initial search strategies were validated through pilot tests to assess the retrieval of previously identified relevant studies and to adjust the search terms used. To identify additional relevant evidence, the reference lists of included studies were manually reviewed. The complete search strategies are detailed in Supplementary Tables S2–S7.

### 2.2 Study selection

The records identified in the various information sources were imported into the Zotero reference manager (16) for duplicate removal. Unique records were imported into the Rayyan electronic platform (17), where six authors (AA, GAG, RC, KCQ, CDP, GRL) independently assessed compliance with eligibility criteria through title and abstract screening, followed by confirmation of eligibility of previously selected references through full-text reading. The specific criteria used for these stages are detailed in the following section (2.3 Eligibility criteria). In case of studies not available in full text, attempts were made to access them by personal communication with the authors of the studies. Discrepant decisions were resolved through initial consensus among the evaluators or with the participation of all authors.

### 2.3 Eligibility criteria

The inclusion criteria were as follows: (a) population: participants of any age and health condition; (b) intervention: all interventions aimed at modifying (increasing or reducing) UPFs consumption, which could include diets or foods provided by the researchers, nutritional counseling, or educational interventions; (c) control: usual consumption of UPFs or with less intensive modification than the intervention group; (d) outcomes: primary or secondary prevention of non-communicable diseases, modification of risk factors for non-communicable diseases, obstetric, prenatal, or perinatal outcomes, adverse events, and quality of life; (e) study design: RCTs. Studies published in abbreviated formats such as editorials or conference abstracts, or written in languages other than English, Spanish, or Portuguese, were excluded.

### 2.4 Data extraction

Data extraction was independently conducted by six authors (AA, GAG, RC, KCQ, CDP, GRL) using a predefined structured form designed in Microsoft Excel 2016. The data extraction template included information on the first author’s name, year of publication, country, number and characteristics of participants, characteristics of interventions, and summary of results of interest for the review. Discrepant decisions were resolved through initial consensus among the evaluators or with the participation of all authors.

### 2.5 Data synthesis

Due to the included studies reporting different outcomes and being conducted in different populations, it was not possible to conduct a statistical meta-analysis. Instead, narrative synthesis was employed to integrate and summarize the findings of heterogeneous studies more appropriately. The narrative synthesis was systematically conducted following the recommendations of the Synthesis Without Meta-analysis (SWiM) guidelines (see Supplementary Table S8) (18). Results were planned to be grouped by population type to reduce heterogeneity in the analysis and improve the understanding of potential differences in effect between groups. The results were reported using the original effect measures employed in the studies because only single studies were identified for each population type. The synthesis of overall results used the vote-counting method based on the direction of effect due to inconsistency in the effect measures reported across studies (19). In cases where studies reported results at multiple time points, the longest follow-up period was chosen. The results for each outcome and population type were summarized in evidence profile tables using the GRADEpro tool (20).

### 2.6 Risk of bias

Two authors (AA, GAG) independently assessed the risk of bias of the included studies using the revised Cochrane Risk of Bias Tool for Randomized Trials (RoB2) (21). This tool assesses five domains: randomization process, deviations from intended interventions, missing outcome data, measurement of the outcome, and selection of the reported result. In each domain, a series of “signaling questions” are provided, and based on the response to these questions, an algorithm provides a proposed judgment on the risk of bias arising from each domain. Thus, each domain was classified as high risk of bias, low risk of bias, or some concerns. Subsequently, the overall risk of bias for each study was calculated, considered high, if at least one domain was rated as high risk; moderate, if at least one domain had some concerns and none was rated as high risk; or low, if all domains were rated as low risk. Discrepancies were resolved through initial consensus between the assessors or with the participation of all authors. No study was excluded based on the risk of bias assessment. The results of the risk of bias assessment were considered in the analysis of evidence certainty, discussed narratively in the text, and considered when formulating the study’s overall conclusions (22).

### 2.7 Certainty of evidence

The certainty of evidence for each outcome was assessed using the GRADE (Grading of Recommendations Assessment, Development and Evaluation) methodology, employing the GRADEpro GDT tool (23). For randomized controlled trials, initially, high certainty of evidence was considered and then downgraded based on the presence of risk of bias, inconsistency, imprecision, indirectness, and publication bias (24). The certainty of evidence was classified as high, moderate, low, or very low (24). The specific criteria for downgrading the levels of certainty of evidence are detailed in Supplementary Table S9. The initial assessment was conducted by one author (AA) and reviewed by a second author (GAG). Both authors were trained and had extensive experience using the GRADE methodology. Discrepancies were resolved through initial consensus between the assessors or with the participation of all authors.

## 3 Results

**Figure 1:**
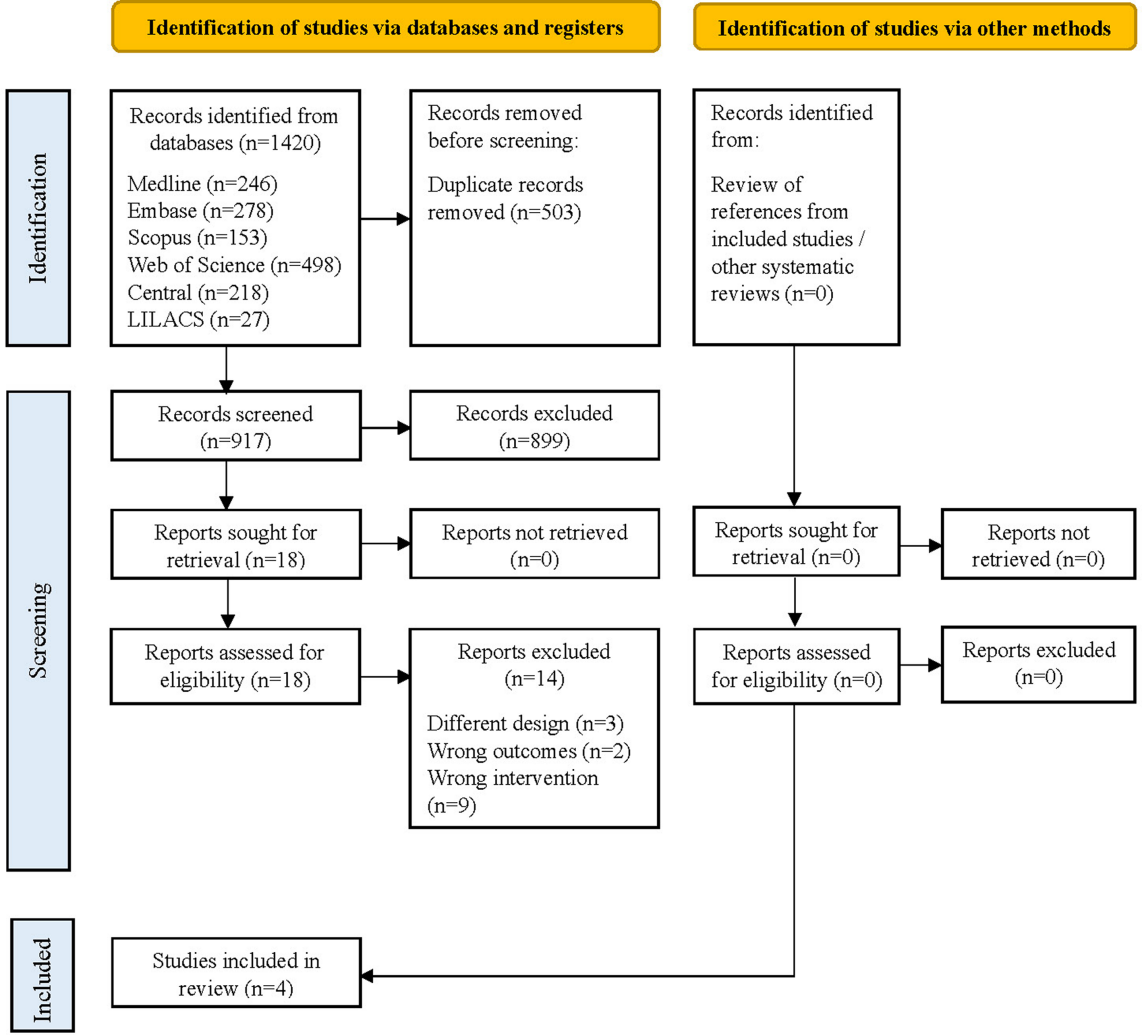
Flow diagram of the study selection process according to PRISMA 2020.

The study selection process is summarized in Figure 1. The literature search identified 1,420 initial records. After removing duplicates, 917 titles and abstracts were reviewed, of which 18 were assessed in full text. Finally, four studies (25–28) met the eligibility criteria and were included in the present systematic review. The reasons for exclusion at full text are detailed in Supplementary Table S10.

### 3.1 Characteristics of included studies

Three studies offered educational interventions (26, 27) or personalized nutritional counseling (25) with recommendations to avoid (25, 26) or limit the consumption of UPFs to less than once a week (27), along with additional recommendations on diet and physical activity. One study corresponded to a controlled feeding trial in which researchers provided participants with an ultra-processed diet and an unprocessed diet to be consumed *ad libitum* in random two-week periods (28). The studies were conducted in Brazil (25, 26), China (27) and the United States (28), and were published between the years 2021 and 2023. The median duration was 12 weeks (range: 4–18 weeks). Three clinical trials employed parallel groups (25–27), while one clinical trial was crossover (28). The studies were conducted in different populations, including overweight pregnant women (25), women with grade I-II obesity (26), children and adolescents with overweight or obesity (27), and adults with stable weight (28) (Tables 1, 2).

**Table 1:** Characteristics of included randomized controlled trials (RCTs).

Author, year (reference)	Type of RCT (registry)	Country	Setting	n	Duration (weeks)	Population	Type of intervention	Funding
Sartorelli (25)	Parallel(RBR-2w9bhc)	Brazil	Outpatient	335	≈18^*^	Overweight pregnant women Age (median): 27 y Gestational age (median): 11 weeks BMI, kg/m^2^ (median): IG: 27.2 | CG: 26.9 Race/ethnicity: mulatto (IG: 53% | CG: 53.9%)	Nutritional counselling	Public
Giacomello (26)	Parallel (Not reported)	Brazil	Outpatient	40	12	Women with grade I-II obesity Age (mean): IG: 35.75 y | CG: 34.75 y BMI, kg/m^2^ (mean): IG: 35.3 | CG: 34.1 Race/ethnicity: caucasian (IG: 80% | CG: 75%)	Educational intervention	Public
Chen (27)	Parallel (ChiCTR190002174)	China	Outpatient	60	12	Children and adolescents with overweight or obesity Age (mean): 10.8 y BMI, kg/m^2^ (mean): 27.0 Women: 29.6%	Educational intervention	Public
Hall (28)	Cross-over (NCT03407053)	United States	Hospital	20	4^a^	Weight-stable adults Age (mean): 31.2 y BMI, kg/m^2^ (mean): 27.0 Women: 50%	Diets provided by the researchers	Public

^*^Follow-up between week 16 or earlier and weeks 34–36 of gestation. BMI, body mass index; CG, control group; IG, intervention group; y, years old.

a Two consecutive dietary periods of 2 weeks each.

**Table 2:** Main characteristics of the interventions.

Author, year (reference)	Intervention	Control
Sartorelli (25)	No. (initial/end): 169/124 Three 30-min sessions offered between the second trimester and weeks 34–36 of gestation, delivered by trained nutritionists. Recommendation on UPFs: avoid Other recommendations: adequate weight gain, daily intake of fruits and vegetables, 150 min/week of physical activity Co-intervention: routine prenatal care.	No. (initial/end): 166/143 Nutritional counseling as part of routine prenatal care: one session focused on assessing nutritional status, healthy weight gain, detection of potential nutritional deficiencies, and clarification of misconceptions, delivered by nurses. Recommendation on UPFs: none Co-intervention: routine prenatal care.
Giacomello (26)	No. (initial/end): 20/14 Educational intervention, expository dialogue type. Five biweekly 90-min group meetings offered over a three-month period. Recommendation on UPFs: avoid Other recommendations: natural or minimally processed foods as the basis of the diet; use of oils, fats, salt, and sugar in small amounts; limit the use of processed foods.	No. (initial/end): 20/10 No intervention.
Chen (27)	No. (initial/end): 30/29 Educational intervention: one session delivered by a registered nutritionist, providing printed material on allowed and forbidden foods, a reference weekly menu model, and telephone support every 3 weeks, for 12 weeks. Recommendation on UPFs: <1 serving/week Other recommendations: no caloric restriction, moderate reduction of starchy foods, 60 min/day of moderately vigorous physical activity.	No. (initial/end): 30/25 Delivery method similar to that of the intervention group. Recommendation on UPFs: reducing frequent consumption (except packaged breads, steamed buns and dumplings). Other recommendations: intake of 1,100 to 2,300 kilocalories (by age), reducing frequent consumption of sugar-sweetened beverages, 60 min/day of moderately vigorous physical activity.
Hall (28)	No. (initial/end): 20/20 Participants were randomly assigned to receive two interventions (ultra-processed and unprocessed diets) in a random sequence, each lasting 2 weeks. The researchers provided three main meals (breakfast, lunch, and dinner) along with snacks available throughout the day. Participants were instructed to consume as much as they desired (*ad libitum*) during each mealtime for 60 min. Ultra-processed diet period Energy from UPFs: 81.3%	No. (initial/end): 20/20 Unprocessed diet period Energy from UPFs: 0% Diet designed to provide the same calories, energy density, macronutrients, sugar, sodium, and fiber as the UPF diet.

### 3.2 Summary of findings

A general summary of the findings is presented in Table 3.

**Table 3:** Summary of findings.

Description of the interventions	Outcome	Effects of the intervention	GRADE evidence^*^
**Overweight pregnant women** (25)			
**Intervention:** Nutritional counselling (avoid UPFs + gestational weight gain + intake of fruits and vegetables + physical activity) **Control:** Regular nutritional counseling (without recommendations on UPFs).	Excessive gestational weight gain	Reduction^a^	⨁◯◯◯ Very low
	Adequate gestational weight gain	No effect	⨁◯◯◯ Very low
	Insufficient gestational weight gain	No effect	⨁◯◯◯ Very low
	Gestational hypertension	No effect	⨁◯◯◯ Very low
	Gestational diabetes mellitus	No effect	⨁◯◯◯ Very low
	Preterm birth	No effect	⨁◯◯◯ Very low
	Caesarean delivery	No effect	⨁◯◯◯ Very low
	Preeclampsia	No effect	⨁◯◯◯ Very low
**Women with grade I-II obesity** (26)			
**Intervention:** Educational intervention (avoid UPFs + other dietary recommendations) **Control:** No intervention	Systolic blood pressure	No effect	⨁◯◯◯ Very low
	Diastolic blood pressure	No effect	⨁◯◯◯ Very low
	HDL cholesterol	No effect	⨁⨁◯◯ Low
	Triglycerides	No effect	⨁⨁◯◯ Low
	Body weight	Reduction^b^	⨁⨁◯◯ Low
	Hip circumference	Reduction^b^	⨁⨁◯◯ Low
	Waist circumference	Unclear^c^	⨁⨁◯◯ Low
	Quality of life - Domain: pain	No effect	⨁◯◯◯ Very low
	Quality of life - Domain: physical aspects	No effect	⨁◯◯◯ Very low
	Quality of life - Domain: general health status	No effect	⨁◯◯◯ Very low
	Quality of life - Domain: vitality	Unclear^c^	⨁◯◯◯ Very low
	Quality of life - Domain: mental health	Unclear^c^	⨁◯◯◯ Very low
	Quality of life - Domain: functional capacity	Improvement^b^	⨁◯◯◯ Very low
	Quality of life - Domain: social aspects	Improvement^b^	⨁◯◯◯ Very low
	Quality of life - Domain: emotional aspects	Improvement^b^	⨁◯◯◯ Very low
**Children and adolescents with overweight or obesity** (27)			
**Intervention:** Educational intervention (avoid UPFs: <1 serving/week + no caloric restriction + moderate reduction of starchy foods + physical activity) **Control:** Educational intervention (reduction of UPFs + caloric restriction + reducing frequent consumption of sugar-sweetened beverages + physical activity)	Body mass index	No effect	⨁⨁⨁◯ Moderate
	Fasting glucose	No effect	⨁⨁⨁◯ Moderate
	Fasting insulin	No effect	⨁⨁⨁◯ Moderate
	Total cholesterol	No effect	⨁⨁⨁◯ Moderate
	Triglycerides	No effect	⨁⨁⨁◯ Moderate
	Serum uric acid	No effect	⨁⨁⨁◯ Moderate
	Fat mass percentage	No effect	⨁⨁⨁◯ Moderate
**Weight-stable adults** (28)			
**Intervention:** Ultra-processed diet *ad libitum* (breakfast, lunch, and dinner). Energy from UPFs: 81.3% **Control:** Unprocessed diet *ad libitum* (breakfast, lunch, and dinner). Energy from UPFs: 0%	Energy intake	Reduction	⨁⨁◯◯ Low
	Carbohydrate intake	Reduction	⨁⨁◯◯ Low
	Fat intake	Reduction	⨁⨁◯◯ Low
	Protein intake	No effect	⨁⨁◯◯ Low
	Body weight	Reduction	⨁⨁⨁◯ Moderate
	Glucose	No effect	⨁⨁⨁◯ Moderate
	Insulin	No effect	⨁⨁⨁◯ Moderate
	Glycated hemoglobin	No effect	⨁⨁⨁◯ Moderate
	Total cholesterol	Reduction	⨁⨁⨁◯ Moderate
	HDL cholesterol	Reduction	⨁⨁⨁◯ Moderate
	LDL cholesterol	No effect	⨁⨁⨁◯ Moderate
	Triglycerides	No effect	⨁⨁⨁◯ Moderate

^*^For further details, please refer to Supplementary Tables S11–S14.

a The intervention did not reduce the intake of UPFs, so the benefits would be attributable to other components of the intervention.

b Significant reduction or improvement in the intervention group, with no changes in the control group. Results were not compared between the groups.

c Significant reduction in both the intervention group and the control group. Results were not compared between the groups.

#### 3.2.1 Overweight pregnant women

A clinical trial (25) assessed the effects of an intervention based on three personalized nutrition counseling sessions, each lasting 30 min, provided by trained nutritionists between the second trimester and weeks 34–36 of gestation. The recommendations included avoiding UPFs consumption, consuming fruits and vegetables daily and engaging in 150 min of physical activity per week. Dietary intake was assessed using food frequency questionnaires, which were not validated for use in pregnant women. The NOVA system was used to classify foods as ultra-processed. However, no actions to ensure the reliability and validity of the assigned classification were mentioned.

The intervention did not produce significant differences in terms of adequate or insufficient gestational weight gain, gestational hypertension, gestational diabetes, premature birth, cesarean delivery, or preeclampsia compared to a control group that received standard nutritional counseling without specific recommendations regarding UPF consumption. A lower likelihood of excessive gestational weight gain was observed in the intervention group when modified intention-to-treat analysis was used, excluding those who did not attend any counseling sessions (OR: 0.56; 95% CI: 0.32 to 0.98; *p* = 0.04), but not when conventional intention-to-treat analysis was employed (including all randomized women). It is worth mentioning that the proposed intervention did not significantly reduce UPF consumption in the study population (25).

#### 3.2.2 Women with grade I-II obesity

A clinical trial (26) evaluated the effect of a group educational intervention, administered for 90 min every 2 weeks over a 12-week period. The recommendations included avoiding UPFs consumption, prioritize the consumption of natural or minimally processed foods, and limit the intake of fats, salt, and sugar, compared to a control group that received no intervention. Food consumption was assessed using a 24-h recall. The classification of UPFs was developed by a registered nutritionist based on the guidelines from the Brazilian Ministry of Health, which are supported by the NOVA classification system. However, it is not described whether additional strategies were considered to ensure the validity and reliability of the assigned classification.

The authors analyzed changes within each group, without comparing differences between the groups. No changes were observed in systolic or diastolic blood pressure, HDL cholesterol, and triglycerides in either group. A significant reduction in body weight (*p* < 0.05) and hip circumference (*p* < 0.001) was observed only in the intervention group, and in waist circumference in both groups (*p* < 0.05 in the control group and *p* < 0.001 in the intervention group). Regarding quality of life assessed with the SF-36 questionnaire, no changes were observed in the dimensions of pain, physical aspects, and general health status in either group. Both groups showed significant improvements in vitality and mental health (*p* < 0.05). Significant changes were observed only in the intervention group for the dimensions of functional capacity, social aspects, and emotional aspects (*p* < 0.05 for functional capacity and *p* < 0.01 for the other two dimensions). UPF consumption decreased in both the intervention group and the control group that received no intervention.

#### 3.2.3 Children and adolescents with overweight or obesity

A clinical trial (27) evaluated the effect of two educational interventions with different levels of UPFs reduction. In one of them, participants were instructed to follow an intensive reduction in UPFs consumption (less than one serving per week) without caloric restriction. In the second intervention, participants were asked to reduce frequent UPF consumption and limit calorie intake to between 1,100 and 2,300 Kcal, depending on age. Both educational interventions were provided only once at the beginning of the study and were accompanied by general recommendations to engage in moderately vigorous physical activity for 60 min per day. The classification of UPFs was based on the NOVA system and was carried out by a registered dietitian, although details about the additional procedures followed to ensure the validity and reliability of the assigned classification were not provided. To improve the identification of UPFs by the participants, a list of allowed and prohibited foods, as well as a reference weekly menu model, was provided.

No significant differences were found between the groups in relation to body mass index, fasting glucose, fasting insulin, total cholesterol, triglycerides, serum uric acid, or fat mass. The study did not assess changes in dietary intake or UPF consumption.

#### 3.2.4 Adults with stable weight

A crossover clinical trial (28) provided participants with either ultraprocessed or minimally processed diets in random sequences of two weeks each. Participants were admitted to a clinical research unit for the entire study duration. They were offered three daily meals with instructions to eat as much as they desired for up to 60 min per meal. The selection of foods and beverages for each diet (ultra-processed or minimally processed) was based on the NOVA classification system. However, no details were provided regarding who conducted the selection or the procedures employed to ensure the validity and reliability of the assigned classification.

Energy consumption was significantly higher during the ultra-processed diet (mean difference [MD]: 508 ± 106 kcal/day, *p* = 0.0001), with a higher intake of carbohydrates (MD: 280 ± 54 kcal/day, *p* = 0.0001) and fats (MD: 230 ± 53 kcal/day, *p* = 0.0004), but not of protein. Participants gained 0.9 ± 0.3 kg during the ultra-processed diet (*p* = 0.009) and lost 0.9 ± 0.3 kg during the unprocessed diet (*p* = 0.007). Fat mass increased by 0.4 ± 0.1 kg during the ultra-processed diet (*p* = 0.0015), with no significant changes during the unprocessed diet. No significant differences were observed at the end of each dietary period in glucose, insulin, or glycated hemoglobin. A greater reduction in total cholesterol and HDL was observed after the unprocessed diet period (*p* = 0.001 and *p* < 0.0001, respectively), with no differences in LDL cholesterol and triglycerides.

### 3.3 Risk of bias

**Figure 2:**
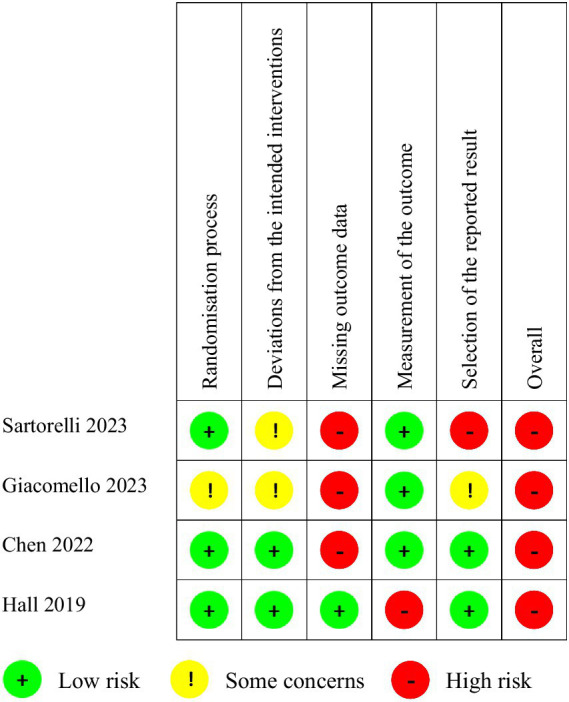
Summary of risk of bias.

All studies had a high risk of bias (Figure 2). Three studies (25–27) reported losses to follow-up exceeding 20% of participants and were considered at high risk of bias due to missing outcome data. Additionally, one study (25) was deemed to have a high risk of bias in the selection of the reported result because the reported primary outcome (excessive gestational weight gain) differed from the planned protocol (adequate gestational weight gain). Moreover, some concerns were identified regarding deviations from the planned interventions in two studies (25, 26), as well as in the randomization process and selection of the reported result in one study (26). These concerns arose due to insufficient information provided in the studies. Finally, the controlled feeding trial (28) presented a high risk of outcome measurement bias due to the lack of blinding of participants, which could have influenced food consumption in each dietary period, and of outcome assessors, which could have influenced outcome assessment.

### 3.4 Certainty of evidence

The certainty of evidence using the GRADE system was considered very low for the outcomes of interest in the population of overweight pregnant women (25); low or very low in women with obesity (26); moderate in children and adolescents with overweight or obesity (27); and low or moderate in adults with stable weight (28) (Table 3 and Supplementary Tables S11–S14).

## 4 Discussion

The present systematic review aimed to examine the evidence derived from RCTs regarding the health effects of UPFs consumption. Four studies were identified, reporting a total of 42 distinct outcomes. Reduction in UPFs consumption resulted in improvements in some anthropometric outcomes and others related to dietary intake, although most outcomes evaluated did not show significant effects. The studies included in the review comprised one controlled feeding trial that provided participants with specially designed diets (28) and three studies based on educational interventions (25–27).

With regard to studies based on educational interventions, it is important that they can achieve an effective reduction in UPFs consumption to attribute their results to such interventions. However, only two of these studies evaluated final changes in UPFs intake (25, 26). In one study, the proposed educational intervention failed to reduce UPFs consumption (25), while in the remaining study, the control group that did not receive any intervention showed a reduction in UPFs intake similar to the intervention group (26). Hence, in both studies, the observed benefits could be attributed to other dietary and physical activity components included in the interventions.

The low adherence to dietary recommendations is a common issue that can affect 30–50% of participants in clinical trials, particularly in long-term interventions or those employing strict elimination diets (29–31). In the study conducted by Sartorelli et al., participants were from areas of high social vulnerability, which, according to the authors, could have limited their access to a healthy diet (25). There is evidence that the consumption of UPFs is usually higher in disadvantaged populations due to greater availability, access, and lower cost (5, 32). In fact, according to the study by Hall (28), the weekly cost to prepare a diet of 2000 Kcal/day consisting of ultra-processed foods was US$ 106, compared to US$ 151 for an unprocessed diet. Therefore, it has been suggested that policies to reduce UPF intake take into account the most vulnerable groups to avoid deepening economic, health, and social inequalities (33). Strategies have been proposed, such as including these groups in food assistance programs to improve access to natural and minimally processed foods (2), as well as reformulating UPFs. However, this latter approach has been criticized by those who argue that it could legitimize and encourage the consumption of these types of products (34).

On the other hand, studies based on educational interventions recorded significant dropout rates. In two studies, dropouts reached between 20 and 40% of participants (25, 26), while in the remaining study, although only 10% dropouts were reported, these were five times more frequent in the control group (27). High rates of overall or differential dropout represent a significant concern in dietary clinical trials (31). Although they are often observed in long-term interventions, dropouts exceeding 50% have been reported even in dietary interventions of only 12 weeks (35). This situation can lead to missing data, biases, loss of statistical power, and compromise the integrity of the randomization process, compromising the validity of the results and their conclusions (31, 35).

To reduce dropout rates, several strategies have been proposed, such as increasing participant recruitment; including a *run-in* period that allows participants to assess the required commitment and researchers to identify those with low motivation, poor commitment, or limited availability; offering a flexible nutritional intervention as much as possible; maintaining regular contact with participants to monitor compliance, provide counseling, and strengthen the relationship with researchers; highlighting the benefits and positive aspects of their participation in the study; and offering financial incentives (36–38).

In contrast to the high dropouts rates observed in trials based on educational interventions, the controlled feeding trial conducted by Hall et al. (28) did not register any dropouts. In these types of studies, participants do not face obstacles related to the acquisition and preparation of food, which could favor their retention in the study and ensure compliance with the dietary intervention protocol (39). Although these types of studies allow hypotheses to be tested under highly controlled conditions (40), they may present limitations related to sample size, study duration, and the resources required to carry them out. Controlled feeding trials are costly, as they require highly trained and certified personnel, equipment for food preservation and storage, special facilities, laboratory capabilities to analyze the nutritional composition of diets, objective tests to determine daily energy needs, access to nutritional biomarkers or monitoring devices, transportation and logistics, in addition to the cost of food supply (around US$30 per participant per day) (40, 41).

Furthermore, these studies are usually of short duration not only due to costs but also because participants face a substantial burden that translates into significant changes in their daily routines and lifestyle, making adherence more difficult over longer periods (40, 42). Additionally, this type of study often enrolls highly motivated individuals who may have very different characteristics from the general population (42). Finally, sample sizes tend to be small, around 5–25 participants, as larger sample sizes may require multiple study sites and increase associated costs (41).

In the controlled feeding trial by Hall et al. (28), the researchers designed two different diets in the percentage of calories derived from UPFs, but similar in total calories, energy density, macronutrients, total fiber, sugars, and sodium. Some methodological aspects to consider include differences between the diets in the content of added sugar, insoluble fiber, saturated fat, and the omega 3:6 ratio that could interfere with the observed results (14). Additionally, equivalence in total fiber content was achieved by including dissolved fiber supplements in beverages in the ultra-processed diet, which could have a lesser impact on satiety (28). There was also no adaptation period employed to control for initial exposure (40), nor an elimination period to avoid carry-over effects from previous interventions (43). However, in a secondary analysis of the same study, no carry-over effects on energy intake, weight, or body composition were identified (44).

It’s also important note that all studies included in the review employed the NOVA system (not an acronym) to classify foods as ultra-processed. This system, first proposed in 2009, has been widely used in academic publications and national and international dietary guidelines (45). However, its use has not been without controversy. A primary point of contention relates to the value it could add to existing classification systems based on diet quality or nutrient profile (46, 47). Opponents argue that the association between UPFs and adverse health outcomes could be explained solely by an unfavorable nutritional profile (32). However, a review of prospective studies showed that the majority of associations between UPFs and health-related outcomes remained significant and unchanged in magnitude after adjustment for diet quality, suggesting that increased consumption of UPFs could produce negative effects independent of their nutritional composition (48).

It is also argued that the category of UPFs encompasses a wide variety of products and ingredients that could have different effects on health (1, 32). For example, one study demonstrated that excluding foods with more than 25% whole grains from the classification of UPFs did not alter the association between UPFs and cardiometabolic risk factors (49). Additionally, certain foods such as dark chocolate or yogurt, predominantly considered as UPFs, have been associated with cardiovascular and cognitive health benefits, as well as a reduced risk of diabetes and colorectal cancer (50–53). It is important to note that the health risks of these foods likely differ from other types of UPFs, such as sugary beverages or processed meats, thus necessitating a more precise differentiation of health effects among different types of UPFs (46).

On the other hand, some authors argue that the NOVA system is based on a descriptive classification approach that can lead to ambiguity, difficulties in interpretation, and produce imprecise and inconsistent judgments among evaluators (33, 54). A study conducted in France, where food and nutrition specialists were asked to assign foods to NOVA categories, revealed low overall consistency among evaluators (55). Conversely, a study conducted in the United States found an 88.3% agreement level among evaluators, although the authors argue that this precision may not be sufficient to draw appropriate inferences about UPF consumption from a single evaluator (56). Additional studies are required to identify strategies to improve consistency in assignments among different evaluators when using the NOVA system. This could enhance the reliability of conclusions drawn from studies employing this classification, as well as their ability to guide public health policies and inform consumers (55).

Similarly, it’s important to note that the causal mechanisms linking UPF consumption to disease risk are not yet fully understood (32). One proposed mechanism links UPFs to a general deterioration in diet quality due to excessive amounts of calories, added sugars, refined grains, unhealthy fats, and sodium present in their composition, as well as the potential to displace or substitute healthy unprocessed or minimally processed foods (32, 46). However, it has also been suggested that UPFs could be harmful to health due to the level of industrial processing they undergo.

For example, the extrusion and retrogradation of starch affect the availability of carbohydrates, while particle size and viscosity influence glycemic response, whose postprandial level can impact appetite and fat storage in the liver and skeletal muscle, which is associated with the development of insulin resistance (47, 57). Furthermore, ultra-processing modifies the texture of foods, making them softer and easier to consume, promoting continuous and unconscious eating behaviors (46), and a faster energy intake that affects satiety, transit time, digestibility, and nutrient bioavailability (2, 33). These relationships could be due to inadequate signaling of satiety sensations to the brain, the release of hormones that regulate hunger and satiety, and a shorter gastric emptying time that increases the speed at which nutrients are released and absorbed in the body (58, 59).

UPFs can also influence homeostatic mechanisms of body weight regulation (33) and create an intestinal environment conducive to the proliferation of microorganisms that promote inflammatory diseases (46). Additionally, industrial processing can generate potentially toxic compounds, such as furans, heterocyclic amines, polycyclic aromatic hydrocarbons, acrolein, advanced glycation end products, industrial trans fatty acids, and acrylamide, associated with an increased risk of chronic diseases (32, 46). These risks have been attributed to increased inflammatory mechanisms, alteration of intestinal barrier function, changes in the microbiota, among others (60–62). Lastly, UPFs often have an extended shelf life, which could facilitate the migration of contaminants from their packaging, such as phthalates, bisphenols, mineral oils, and microplastics (32). These substances can increase the risk of cardiovascular diseases, obesity, insulin resistance, type 2 diabetes and cancer by altering normal hormonal activity and activating nuclear receptors such as PPAR α, β y γ, and the retinoid X receptor, which control various aspects of energy metabolism, inflammation, and cellular homeostasis (63, 64).

Although numerous mechanisms beyond their nutritional content have been proposed to explain the relationship between UPFs and adverse health effects, these mechanisms have not been fully elucidated (32, 47). Currently, there is no single plausible explanation for a common effect of all UPFs on the various health effects reported in the literature (65). Therefore, further research is needed to better understand how UPFs relate to these adverse outcomes (66).

In our knowledge, this is the first systematic review that evaluates the effects of UPF consumption based on evidence from randomized clinical trials. Previously, numerous reviews analyzed the health effects of UPF consumption based on evidence from observational studies. However, reviews of observational studies have limitations, such as the inclusion of studies that use different methods to assess UPF intake (67), different models for adjusting covariates (67, 68), heterogeneous doses of intake between higher and lower exposure groups (1, 68), different classification systems and reference units (1, 46), as well as variable follow-up periods (69). Therefore, considering the growing importance of UPFs in shaping global nutrition policies and guidelines, high-quality clinical trials are needed to overcome these limitations and define causality mechanisms that cannot be solely inferred from observational studies (33).

However, it is important to consider that designing long-term clinical trials of dietary interventions is not always feasible or ethical, especially when seeking to demonstrate the effect of interventions with potential risks, such as diets high in UPFs (32, 67). Therefore, it is more feasible to expect short-term trials that evaluate their impact on intermediate outcomes (67). These intermediate outcomes correspond to surrogate markers, such as changes in physiologic measures, that can infer or predict clinically relevant outcomes for patients, such as death or quality of life (70, 71). Although these outcomes are widely used in clinical trials, their validity requires demonstrating a strong association with the final outcomes they aim to substitute (70). However, for many intermediate outcomes, there are considerable doubts about their correlation with final outcomes (70). For example, reducing the intake of saturated fats has a favorable impact on lipid profile and anthropometric measures, although it has not demonstrated a clear association with cardiovascular mortality (72).

In this context, demonstrating causality represents a significant challenge, for which triangulation has been proposed as the best approach based on integrating evidence from multiple study designs, such as short-term trials, mechanistic studies, and well-conducted large-scale epidemiological observational studies (47, 73–75). Corroborating different types of evidence can yield more robust dietary guidelines and better inform causal inference on complex issues that cannot be directly studied (47).

Our review had several strengths, among which we can highlight being the first review aimed at examining the association between UPF consumption and health-related outcomes based on RCT evidence. Additionally, we conducted a comprehensive literature search, analyzed a wide variety of outcomes in diverse populations, and adhered to strict methodological standards such as the guidelines established by the PRISMA statement and the SWiM guidelines to ensure transparent reporting, the methodological guidelines provided by the Cochrane Collaboration for conducting systematic reviews of interventions, and the GRADE system to evaluate of the certainty of evidence.

GRADE is a system adopted by over 120 organizations worldwide, which provides a framework for systematic and transparent evaluation of evidence certainty, including an explicit record of judgments made (23, 76). However, some weaknesses have been pointed out, such as variability in judgments obtained by different evaluators (77). Taking this into consideration, the assessment was conducted by two authors trained and experienced in using the GRADE methodology, as this approach has been shown to enhance result reliability (78). Furthermore, it has been suggested that GRADE may not fully align with the specific requirements of nutrition research, which has prompted the development of alternative systems based on adaptations to GRADE (79), although further investigation is still required to validate their effectiveness.

We must also acknowledge some limitations. We were unable to synthesize the findings through meta-analysis due to the heterogeneity in the characteristics of the included studies (80, 81), and, instead, we employed a narrative synthesis. It is estimated that between 32 to 56% of systematic reviews used this type of synthesis (18, 80). Nonetheless, its use may face issues related to insufficient reporting of the methods employed and their limitations, which could affect the validity of the review findings (18, 81). To minimize these risks, we followed the recommendations of the Synthesis Without Meta-analysis (SWiM) guidelines (18). However, the limited number of studies and the differences in the characteristics of the populations prevented us from conducting subgroup analysis or limit the analysis to studies with lower risk of bias, which would have allowed for a deeper investigation into the sources of heterogeneity of the observed effects (19).

On the other hand, one of the main methodological deficiencies in systematic reviews on UPFs has been the lack of an explicit approach to the risk of bias in primary studies during evidence synthesis (82). Although in our review, we employed GRADE to incorporate the risk of bias into evidence synthesis, discussing the potential effect of deviations from intended interventions and the lack of outcome data, and considering these limitations in the overall study conclusions, we were unable to implement other strategies, such as sensitivity analysis (22), due to the limited number of studies and the different populations in which they were conducted.

Finally, we must also consider the limitations of the primary studies include in our review. In three out of the four included studies, participants only received educational interventions with recommendations to reduce UPFs consumption. In these studies, ensuring effective reduction of UPFs consumption is required for the observed health effects to be attributable to such interventions. However, we observed low adherence and high dropout rates. All these factors, coupled with the limited number of studies, short follow-up periods, high risk of bias, and a generally low or very low certainty of evidence, make it difficult to draw definitive conclusions about the true effect of UPFs on health.

## 5 Policy implications and future research

Around the world, various institutions, including the World Health Organization, have recommended reducing or avoiding the intake of UPFs (67). Some countries already include this recommendation as part of their dietary guidelines or have implemented strategies to reduce their consumption, such as the use of nutritional warning labels, selective taxes, marketing restrictions, or bans in schools (67). However, these recommendations are primarily based on results from prospective observational studies with methodological limitations and not designed to establish causal mechanisms (33). This reliance on observational study results to support nutritional recommendations is common (29) and often reflects the difficulty of developing long-term dietary clinical trials (32, 67). In fact, our review identified only four clinical trials, of which only one directly evaluated the effects of consuming a UPF-based diet, although in a small number of participants, with a short follow-up period and based on intermediate outcomes.

Consequently, the available evidence to date cannot establish a clear causal link between the degree of food processing and adverse health outcomes. Despite the undeniable fact that certain elements commonly present in UPFs, such as salt or sugar, contribute to the development of various chronic diseases (83), the added value of classifying foods based on their industrial processing compared to traditional nutrient-based systems remains an unresolved controversy.

From the perspective of health and nutrition policies, it is necessary for governments to promote measures to encourage the consumption of healthy natural or minimally processed foods, making them more available, valued, and affordable (83). Likewise, it should be recognized that UPFs play a central role in food systems and can be drivers of diet quality in contexts where nutrient-rich foods are scarce or have limited access (33, 83). In this sense, policies to limit UPF consumption should be accompanied by strategies that minimize any negative impact on the food security of vulnerable groups (33), including regulations to facilitate the reformulation of UPFs, especially those aimed at replacing processed, refined, and reconstituted ingredients with intact or minimally processed ingredients (83). Finally, considering that non-communicable diseases are of a multifactorial nature, health, and nutrition policies must be based on comprehensive approaches that address both dietary factors and other social, economic, and environmental determinants, as well as evaluate the impact of the strategies adopted (84).

From a research perspective, establishing the causal mechanisms that link UPFs to adverse health outcomes will require well-designed prospective studies that overcome current methodological limitations, mechanistic studies that identify the specific attributes involved in the pathogenesis of the disease, and a greater number of dietary clinical trials with more participants and longer follow-up periods (67, 83). Similarly, studies aimed at improving the accuracy and consistency of the NOVA system, the most widely used classification system to identify UPFs, will allow for a more reliable and functional system (4, 33). Finally, studies are needed to understand the differences between the various types of UPFs and their effects on human health (4, 33, 67).

## 6 Conclusion

Our findings show that interventions aimed at reducing the consumption of UPFs had beneficial effects on some anthropometric and dietary intake outcomes, although no significant effect was observed for most of the evaluated outcomes. However, due to the limited number of studies and significant methodological limitations identified, we cannot draw definitive conclusions. Further well-designed clinical trials are needed to enhance our understanding of the relationship between UPF consumption and health outcomes to promote effective policies.

## Data availability statement

The original contributions presented in the study are included in the article/Supplementary material, further inquiries can be directed to the corresponding author.

## Author contributions

AA: Conceptualization, Data curation, Formal analysis, Investigation, Methodology, Writing – original draft, Writing – review & editing. GA-G: Data curation, Formal analysis, Investigation, Writing – original draft, Writing – review & editing. RC: Data curation, Formal analysis, Investigation, Writing – original draft, Writing – review & editing. KC-Q: Conceptualization, Data curation, Formal analysis, Investigation, Writing – original draft, Writing – review & editing. CD-P: Data curation, Formal analysis, Investigation, Writing – original draft, Writing – review & editing. GR-L: Data curation, Formal analysis, Investigation, Writing – original draft, Writing – review & editing. CL: Conceptualization, Funding acquisition, Supervision, Writing – original draft, Writing – review & editing.
