# Supplementary material for: Ultra-processed foods consumption and health-related outcomes: a systematic review of randomized controlled trials
Source: Front Nutr. 2024 Jun 26;11:1421728. doi: 10.3389/fnut.2024.1421728 (PMC11233771; doi:10.3389/fnut.2024.1421728)
Supplement: Supplementary file 1 [file Data_Sheet_1.docx]

Supplementary Material

Contents

[Supplementary Table S1. PRISMA Statement Checklist 2](#_Toc168492586)

[Supplementary Table S2. Search strategy in Medline (via Ovid) 5](#_Toc168492587)

[Supplementary Table S3. Search strategy in Embase (via embase.com) 6](#_Toc168492588)

[Supplementary Table S4. Search strategy in Web of Science 7](#_Toc168492589)

[Supplementary Table S5. Search strategy in Scopus. 7](#_Toc168492590)

[Supplementary Table S6. Search strategy in LILACS. 7](#_Toc168492591)

[Supplementary Table S7. Search strategy in CENTRAL. 8](#_Toc168492592)

[Supplementary Table S8. Synthesis Without Meta-analysis (SWiM) Checklist 9](#_Toc168492593)

[Supplementary Table S9. GRADE criteria for assessing the certainty of the evidence. 10](#_Toc168492594)

[Supplementary Table S10. Excluded full texts with reasons. 11](#_Toc168492595)

[Supplementary Tables S11-S14. GRADE assessment. 13](#_Toc168492596)

**Supplementary Data**

Supplementary Table S1. PRISMA Statement Checklist

| **Section/topic** | **Item #** | **Checklist item** | **Reported on page #** |
| --- | --- | --- | --- |
| **TITLE** |  |  |  |
| Title | 1 | Identify the report as a systematic review | 1 |
| **ABSTRACT** |  |  |  |
| Abstract | 2 | As per PRISMA 2020 for Abstracts checklist | 1 |
| **INTRODUCTION** |  |  |  |
| Rationale | 3 | Describe the rationale for the review in the context of existing knowledge. | 2 |
| Objectives | 4 | Provide an explicit statement of the objective(s) or question(s) the review addresses. | 2 |
| **METHODS** |  |  |  |
| Eligibility criteria | 5 | Specify the inclusion and exclusion criteria for the review and how studies were grouped for the syntheses. | 2-3 |
| Information sources | 6 | Specify all databases, registers, websites, organizations, reference lists and other sources searched or consulted to identify studies. Specify the date when each source was last searched or consulted. | 2 |
| Search strategy | 7 | Present the full search strategies for all databases, registers, and websites, including any filters and limits used. | Suppl.  (5-8) |
| Selection process | 8 | Specify the methods used to decide whether a study met the inclusion criteria of the review, including how many reviewers screened each record and each report retrieved, whether they worked independently, and if applicable, details of automation tools used in the process. | 2-3 |
| Data collection process | 9 | Specify the methods used to collect data from reports, including how many reviewers collected data from each report, whether they worked independently, any processes for obtaining or confirming data from study investigators, and if applicable, details of automation tools used in the process. | 2-3 |
| Data items | 10a | List and define all outcomes for which data were sought. Specify whether all results that were compatible with each outcome domain in each study were sought (e.g., for all measures, time points, analyses), and if not, the methods used to decide which results to collect. | 3 |
|  | 10b | List and define all other variables for which data were sought (e.g., participant and intervention characteristics, funding sources). Describe any assumptions made about any missing or unclear information. | 3 |
| Study risk of bias assessment | 11 | Specify the methods used to assess risk of bias in the included studies, including details of the tool(s) used, how many reviewers assessed each study and whether they worked independently, and if applicable, details of automation tools used in the process. | 3 |
| Effect measures | 12 | Specify for each outcome the effect measure(s) (e.g., risk ratio, mean difference) used in the synthesis or presentation of results. | 3 |
| Synthesis methods | 13a | Describe the processes used to decide which studies were eligible for each synthesis (e.g. tabulating the study intervention characteristics and comparing against the planned groups for each synthesis (item #5)). | 3 |
|  | 13b | Describe any methods required to prepare the data for presentation or synthesis, such as handling of missing summary statistics, or data conversions. | 3 |
|  | 13c | Describe any methods used to tabulate or visually display results of individual studies and syntheses. | 3 |
|  | 13d | Describe any methods used to synthesize results and provide a rationale for the choice(s). If meta-analysis was performed, describe the model(s), method(s) to identify the presence and extent of statistical heterogeneity, and software package(s) used. | 3 |
|  | 13e | Describe any methods used to explore possible causes of heterogeneity among study results (e.g. subgroup analysis, meta-regression). | -- |
|  | 13f | Describe any sensitivity analyses conducted to assess robustness of the synthesized results. | -- |
| Reporting bias assessment | 14 | Describe any methods used to assess risk of bias due to missing results in a synthesis (arising from reporting biases). | 3 |
| Certainty assessment | 15 | Describe any methods used to assess certainty (or confidence) in the body of evidence for an outcome. | 3 |
| **RESULTS** |  |  |  |
| Study selection | 16a | Describe the results of the search and selection process, from the number of records identified in the search to the number of studies included in the review, ideally using a flow diagram. | 3-4 |
|  | 16b | Cite studies that might appear to meet the inclusion criteria, but which were excluded, and explain why they were excluded. | Suppl.  (11-12) |
| Study characteristics | 17 | Cite each included study and present its characteristics. | 3-6 |
| Risk of bias in studies | 18 | Present assessments of risk of bias for each included study. | 3,9 |
| Results of individual studies | 19 | For all outcomes, present, for each study: (a) summary statistics for each group (where appropriate) and (b) an effect estimate and its precision (e.g., confidence/credible interval), ideally using structured tables or plots. | 5-6 |
| Results of syntheses | 20a | For each synthesis, briefly summarize the characteristics and risk of bias among contributing studies. | 3-6 |
|  | 20b | Present results of all statistical syntheses conducted. If meta-analysis was done, present for each the summary estimate and its precision (e.g., confidence/credible interval) and measures of statistical heterogeneity. If comparing groups, describe the direction of the effect. | 7-8 |
|  | 20c | Present results of all investigations of possible causes of heterogeneity among study results. | -- |
|  | 20d | Present results of all sensitivity analyses conducted to assess the robustness of the synthesized results. | -- |
| Reporting biases | 21 | Present assessments of risk of bias due to missing results (arising from reporting biases) for each synthesis assessed. | 6 |
| Certainty of evidence | 22 | Present assessments of certainty (or confidence) in the body of evidence for each outcome assessed. | 6-8 |
| **DISCUSSION** |  |  |  |
| Discussion | 23a | Provide a general interpretation of the results in the context of other evidence. | 10 |
|  | 23b | Discuss any limitations of the evidence included in the review. | 11 |
|  | 23c | Discuss any limitations of the review processes used. | 11 |
|  | 23d | Discuss implications of the results for practice, policy, and future research. | 11-12 |
| **OTHER INFORMATION** | | | |
| Registration and protocol | 24a | Provide registration information for the review, including register name and registration number, or state that the review was not registered. | 1-2 |
|  | 24b | Indicate where the review protocol can be accessed, or state that a protocol was not prepared. | 2 |
|  | 24c | Describe and explain any amendments to information provided at registration or in the protocol. | -- |
| Support | 25 | Describe sources of financial or non-financial support for the review, and the role of the funders or sponsors in the review. | 12 |
| Competing interests | 26 | Declare any competing interests of review authors. | 12 |
| Availability of data, code, and other materials | 27 | Report which of the following are publicly available and where they can be found; template data collection forms; data extracted from included studies; data used for all analyses; analytic code; any other materials used in the review. | 12 |

Supplementary Table S2. Search strategy in Medline (via Ovid)

Ovid MEDLINE(R) and Epub Ahead of Print, In-Process, In-Data-Review & Other Non-Indexed Citations and Daily (1946 to present)

Date of last search: April 22, 2024

| No. | Search terms | Results |
| --- | --- | --- |
| 1 | exp food, processed/ | 314 |
| 2 | ((ultra-processed or unhealthy or process*) and (food or foods or diet or diets or meal or meals)).ti. | 5974 |
| 3 | 1 or 2 | 6009 |
| 4 | randomized controlled trial.pt. | 611110 |
| 5 | controlled clinical trial.pt. | 95522 |
| 6 | randomized.ti,ab. | 699479 |
| 7 | placebo.ti,ab. | 254240 |
| 8 | *Clinical Trials as Topic/ | 32325 |
| 9 | randomly.ti,ab. | 432544 |
| 10 | trial.ti,ab. | 803052 |
| 11 | 4 or 5 or 6 or 7 or 8 or 9 or 10 | 1755336 |
| 12 | exp animals/ not exp humans/ | 5213332 |
| 13 | 11 not 12 | 1607382 |
| 14 | 3 and 13 | 311 |

# Supplementary Table S3. Search strategy in Embase (via embase.com)

Database coverage: 1940 to present.

Date of last search: April 22, 2024

| No. | Search terms | Results |
| --- | --- | --- |
| #1 | 'ultra-processed food'/exp OR 'processed food':ti | 2135 |
| #2 | (('ultra processed' OR unhealthy OR process*) NEAR/2 (food OR foods OR diet OR diets OR meal OR meals)):ti | 4121 |
| #3 | #1 OR #2 | 5046 |
| #4 | 'clinical trial'/exp | 1910764 |
| #5 | 'randomized controlled trial'/exp | 820199 |
| #6 | 'randomization'/exp | 99350 |
| #7 | 'single blind procedure'/exp | 54407 |
| #8 | 'double blind procedure'/exp | 218184 |
| #9 | 'crossover procedure'/exp | 77559 |
| #10 | 'placebo'/exp | 418654 |
| #11 | 'randomi?ed controlled trial$' | 1166745 |
| #12 | rct | 64434 |
| #13 | 'random allocation' | 2863 |
| #14 | 'randomly allocated' | 47377 |
| #15 | 'allocated randomly' | 3041 |
| #16 | allocated NEAR/2 random | 971 |
| #17 | 'single blind$' | 63407 |
| #18 | 'double blind$' | 302536 |
| #19 | (treble OR triple) NEAR/2 blind$ | 1846 |
| #20 | placebo$ | 546423 |
| #21 | 'prospective study'/exp | 913687 |
| #22 | #4 OR #5 OR #6 OR #7 OR #8 OR #9 OR #10 OR #11 OR #12 OR #13 OR #14 OR #15 OR #16 OR #17 OR #18 OR #19 OR #20 OR #21 | 3250881 |
| #23 | #3 AND #22 | 434 |
| #24 | #3 AND #22 AND [embase]/lim | 350 |

# Supplementary Table S4. Search strategy in Web of Science

Web of Science Core Collection (2001 to present)

Date of last search: April 22, 2024

| No. | Search terms | Results |
| --- | --- | --- |
| #1 | KP=(ultra-processed food*) | 398 |
| #2 | TI=((('ultra processed' OR unhealthy OR process*) AND (food OR foods OR diet OR diets OR meal OR meals))) | 8065 |
| #3 | #1 OR #2 | 8454 |
| #4 | TS= clinical trial* OR TS=controlled trial* OR TS=random* OR TS=placebo* OR TS=(single blind*) OR TS=(double blind*) OR AB=trial* OR AB=random* OR AB=placebo | 2255974 |
| #5 | #3 AND #4 | 547 |

# Supplementary Table S5. Search strategy in Scopus.

Database coverage: 1788 to present.

Date of last search: April 22, 2024

| No. | Search terms | Results |
| --- | --- | --- |
| #1 | ((TITLE (ultra-processed W/2 food*)) OR (TITLE (("ultra processed" OR unhealthy OR process*) W/2 (food OR foods OR diet OR diets OR meal OR meals)))) AND (ABS ((clinic* W/1 trial*) OR (randomi* W/1 control*) OR (randomi* W/2 trial*) OR (random* W/1 assign*) OR (random* W/1 allocat*) OR (control* W/1 trial) OR placebo* OR (control* W/1 stud*) OR (randomi* W/1 stud*) OR (singl* W/1 blind*) OR (singl* W/1 mask*) OR (doubl* W/1 blind*) OR (doubl* W/1 mask*) OR (tripl* W/1 blind*) OR (tripl* W/1 mask*) OR (trebl* W/1 blind*) OR (trebl* W/1 mask*))) | 169 |

# Supplementary Table S6. Search strategy in LILACS.

Database coverage: 1982 to present.

Date of last search: April 22, 2024

| No. | Search terms | Results |
| --- | --- | --- |
| #1 | (mh:('food, processed' OR 'alimentos industrializados')) OR (ti:((ultra-processed OR unhealthy OR process*) AND (food OR foods OR diet OR diets OR meal OR meals))) OR (ti:(ultra-procesado* OR ultraprocesado*)) AND ( db:("LILACS") AND la:("es" OR "en")) | 38 |

# Supplementary Table S7. Search strategy in CENTRAL.

The Cochrane Central Register of Controlled Trials (Issue 3 of 12, March 2024)

Date of last search: April 22, 2024

| No. | Search terms | Results |
| --- | --- | --- |
| #1 | MeSH descriptor: [Food, Processed] explode all trees | 13 |
| #2 | ((ultra-processed OR unhealthy OR process*) AND (food OR foods OR diet OR diets OR meal OR meals)):ti | 344 |
| #3 | #1 OR #2 | 346 |
| #4 | (randomized OR placebo OR randomly OR trial):ti,ab,kw | 1506182 |
| #5 | MeSH descriptor: [Clinical Trials as Topic] this term only | 40761 |
| #6 | #4 OR #5 | 1519047 |
| #7 | #3 AND #6 | 253 |

Supplementary Table S8. Synthesis Without Meta-analysis (SWiM) Checklist

| **SWiM reporting item** | **Item description** | **Page(s)** | **Other*** |
| --- | --- | --- | --- |
| ***Methods*** | | | |
| **1** Grouping studies for synthesis | 1a) Provide a description of, and rationale for, the groups used in the synthesis (e.g., groupings of populations, interventions, outcomes, study design) | 3 |  |
|  | 1b) Detail and provide rationale for any changes made subsequent to the protocol in the groups used in the synthesis | -- |  |
| **2** Describe the standardized metric and transformation methods used | Describe the standardized metric for each outcome. Explain why the metric(s) was chosen, and describe any methods used to transform the intervention effects, as reported in the study, to the standardized metric, citing any methodological guidance consulted. | 3 |  |
| **3** Describe the synthesis methods | Describe and justify the methods used to synthesize the effects for each outcome when it was not possible to undertake a meta-analysis of effect estimates | 3 |  |
| **4** Criteria used to prioritize results for summary and synthesis | Where applicable, provide the criteria used, with supporting justification, to select the particular studies, or a particular study, for the main synthesis or to draw conclusions from the synthesis (e.g., based on study design, risk of bias assessments, directness in relation to the review question) | 3 |  |
| **5** Investigation of heterogeneity in reported effects | State the method(s) used to examine heterogeneity in reported effects when it was not possible to undertake a meta-analysis of effect estimates and its extensions to investigate heterogeneity | -- |  |
| **6** Certainty of evidence | Describe the methods used to assess certainty of the synthesis findings. | 3 |  |
| **7** Data presentation methods | Describe the graphical and tabular methods used to present the effects (e.g., tables, forest plots, harvest plots). Specify key study characteristics (e.g., study design, risk of bias) used to order the studies, in the text and any tables or graphs, clearly referencing the studies included | 3 |  |
| ***Results*** | | | |
| **8** Reporting results | For each comparison and outcome, provide a description of the synthesized findings, and the certainty of the findings. Describe the result in language that is consistent with the question the synthesis addresses, and indicate which studies contribute to the synthesis | 3-8 |  |
| ***Discussion*** | | | |
| **9** Limitations of the synthesis | Report the limitations of the synthesis methods used and/or the groupings used in the synthesis, and how these affect the conclusions that can be drawn in relation to the original review question. | 11 |  |

# Supplementary Table S9. GRADE criteria for assessing the certainty of the evidence.

| **Decision** | **Risk of bias** | **Inconsistency** | **Indirectness** | **Imprecision** | **Publication bias** |
| --- | --- | --- | --- | --- | --- |
| Not serious  (do not downgrade) | Most studies (or domains in the case of single studies) had a low risk of bias | I^2^ < 50% or single study | All studies met the eligibility criteria | The CI 95% of the association measure does not cross either of the imprecision cutoff points (0.75 or 1.25). | There is no evidence of publication bias based on the assessment of asymmetry in funnel plot or Egger's test, or it was not evaluated due to having fewer than 10 studies. |
| Serious  (Downgrade one level) | Most studies (or domains in the case of single studies) had a moderate risk of bias | I^2^ ≥ 50% and <75% | Outcome other than PICO | The CI 95% of the association measure crosses one of the imprecision cutoff points (0.75 or 1.25) and includes the null effect value | Strong suspicion of publication bias based on the assessment of asymmetry in funnel plot or Egger's test. |
| Very serious (Downgrade two levels) | Most studies (or domains in the case of single studies) had a high risk of bias | I^2^ ≥ 75% | Population or intervention other than PICO | The 95% CI of the association measure crosses both imprecision cutoff points (0.75 and 1.25). |  |

# Supplementary Table S10. Excluded full texts with reasons.

| No | Reference | Reason |
| --- | --- | --- |
| 1 | González-Palacios S, Oncina-Cánovas A, García-de-la-Hera M, Martínez-González MÁ, Salas-Salvadó J, Corella D, Schröder H, Martínez JA, Alonso-Gómez ÁM, Wärnberg J, et al. Increased ultra-processed food consumption is associated with worsening of cardiometabolic risk factors in adults with metabolic syndrome: Longitudinal analysis from a randomized trial. Atherosclerosis (2023) 377:12–23. doi: 10.1016/j.atherosclerosis.2023.05.022 | Wrong intervention. Longitudinal analysis of a clinical trial not aimed at modifying the consumption of ultra-processed foods. |
| 2 | Costa CS, Rauber F, Leffa PS, Sangalli CN, Campagnolo PDB, Vitolo MR. Ultra-processed food consumption and its effects on anthropometric and glucose profile: A longitudinal study during childhood. Nutr Metab Cardiovasc Dis (2019) 29:177–184. doi: 10.1016/j.numecd.2018.11.003 | Wrong intervention. Dietary intervention based on the 10 Steps to Healthy Feeding for Infants, does not include the modification of the consumption of ultra-processed foods. |
| 3 | Cortes C, Brandão JM, Cunha DB, Paravidino VB, Sichieri R. Blood pressure variation and ultra-processed food consumption in children with obesity. Eur J Pediatr (2023) 182:4077–4085. doi: 10.1007/s00431-023-05076-z | Wrong design. Secondary analysis of a clinical trial. The groups assigned to the original interventions are not preserved. |
| 4 | Marques DCS, Ferreira WC, Santos IC, Ryal JJ, Marques MGS, Oliveira FM, Milani RG, Mota J, Valdés-Badilla P, Branco BHM. Impacts of a Multi-Professional Family versus Isolated Intervention on Food Level Processing in Overweight Adolescents: A Randomized Trial. Nutrients (2023) 15:935. doi: 10.3390/nu15040935 | Wrong comparison. Both groups receive the same intervention, except for the participation of a family member in one of the groups. |
| 5 | Migliaretti G, Ame C, Ciullo S, Fontana E, Stura I, Nano E, Laino F, Isoardo G. Metabolic and psychological effects of short-term increased consumption of less-processed foods in daily diets: A Pilot Study. Diabetes Metab (2020) 46:66–69. doi: 10.1016/j.diabet.2019.07.002 | Wrong intervention. The intervention consisted of the purchase of products without food additives (does not meet the definition of ultra-processed food). |
| 6 | Moreira PR, Nunes LM, Giugliani ERJ, Gomes E, Führ J, Neves RO, Belin CHS, Bernardi JR. Complementary feeding methods and introduction of ultra-processed foods: A randomized clinical trial. Front Nutr (2022) 9:1043400. doi: 10.3389/fnut.2022.1043400 | Wrong intervention. The effect of different complementary feeding methods on the introduction of ultra-processed foods was evaluated. |
| 7 | Lazzeri B, Leotti VB, Soldateli B, Giugliani ER, Monteiro CA, Martinez Steele E, Pedrotti LG, Drehmer M. Effect of a healthy eating intervention in the first months of life on ultraprocessed food consumption at the age of 4-7 years: a randomised clinical trial with adolescent mothers and their infants. Br J Nutr (2021) 126:1048–1055. doi: 10.1017/S0007114520004869 | Wrong intervention. The dietary intervention was not aimed at modifying the consumption of ultra-processed foods. |
| 8 | Dioneda B, Healy M, Paul M, Sheridan C, Mohr AE, Arciero PJ. A Gluten-Free Meal Produces a Lower Postprandial Thermogenic Response Compared to an Iso-Energetic/Macronutrient Whole Food or Processed Food Meal in Young Women: A Single-Blind Randomized Cross-Over Trial. Nutrients (2020) 12:2035. doi: 10.3390/nu12072035 | Wrong outcomes. The outcomes of interest for the review are not analyzed. |
| 9 | Phillips NE, Mareschal J, Schwab N, Manoogian ENC, Borloz S, Ostinelli G, Gauthier-Jaques A, Umwali S, Gonzalez Rodriguez E, Aeberli D, et al. The Effects of Time-Restricted Eating versus Standard Dietary Advice on Weight, Metabolic Health and the Consumption of Processed Food: A Pragmatic Randomised Controlled Trial in Community-Based Adults. Nutrients (2021) 13:1042. doi: 10.3390/nu13031042 | Wrong intervention. The study had two parts: in a first observational phase, the association between the consumption of ultra-processed foods and components of metabolic syndrome was evaluated. The second part compared the metabolic benefits of 12-hour fasting vs. standard dietary counseling. |
| 10 | Poll FA, Miraglia F, D’avila HF, Reuter CP, Mello ED. Impact of intervention on nutritional status, consumption of processed foods, and quality of life of adolescents with excess weight. J Pediatr (Rio J) (2020) 96:621–629. doi: 10.1016/j.jped.2019.05.007 | Wrong design. It is not a randomized clinical trial. |
| 11 | Teo PS, Lim AJ, Goh AT, R J, Choy JYM, McCrickerd K, Forde CG. Texture-based differences in eating rate influence energy intake for minimally processed and ultra-processed meals. Am J Clin Nutr (2022) 116:244–254. doi: 10.1093/ajcn/nqac068 | Wrong outcomes. The outcomes of interest for the review are not analyzed. |
| 12 | Pagoldh M, Eriksson A, Heimtun E, Kvifors E, Sternby B, Blomquist L, Lapidus A, Suhr O, Lange S, Karlbom U, et al. Effects of a supplementary diet with specially processed cereals in patients with short bowel syndrome. Eur J Gastroenterol Hepatol (2008) 20:1085–1093. doi: 10.1097/MEG.0b013e328303c00a | Wrong intervention. The study evaluated the effects of a diet supplemented with specially processed cereals (does not meet the definition of ultra-processed food). |
| 13 | Valmorbida JL, Baratto PS, Leffa PS, Sangalli CN, Silva JA, Vitolo MR. Consumption of ultraprocessed food is associated with higher blood pressure among 6-year-old children from southern Brazil. Nutr Res (2023) 116:60–68. doi: 10.1016/j.nutres.2023.05.012 | Wrong design. Secondary analysis of a clinical trial. The groups assigned to the original interventions are not preserved. |
| 14 | Brandão LEM, Popa A, Cedernaes E, Cedernaes C, Lampola L, Cedernaes J. Exposure to a more unhealthy diet impacts sleep microstructure during normal sleep and recovery sleep: A randomized trial. Obesity (Silver Spring) (2023) 31:1755–1766. doi: 10.1002/oby.23787 | Wrong intervention. Dietary intervention aimed at modifying fat and sugar content. It does not evaluate the effect of modifying ultra-processed foods. |
| 15 | Brandão JM, Sichieri R, Paravidino VB, Ribas SA, Cunha DB. Treatment of childhood obesity based on the reduction of ultra-processed foods plus energy restriction: A randomised controlled trial based on the Brazilian guidelines. Clin Obes (2024) doi: 10.1111/cob.12648 | Wrong comparison. Both groups receive dietary intervention to reduce UPF consumption. |

# Supplementary Tables S11-S14. GRADE assessment.

**Supplementary Table S11:** GRADE assessment (Studies in pregnant women)

**Bibliography:** Sartorelli DS, Crivellenti LC, Baroni NF, de Andrade Miranda DEG, da Silva Santos I, Carvalho MR, de Lima MC, Carreira NP, Chaves AVL, Manochio-Pina MG, et al. Effectiveness of a minimally processed food-based nutritional counselling intervention on weight gain in overweight pregnant women: a randomized controlled trial. Eur J Nutr (2023) 62:443–454. doi: 10.1007/s00394-022-02995-9

| **Certainty assessment** | | | | | | | **Summary of findings** | | | | |
| --- | --- | --- | --- | --- | --- | --- | --- | --- | --- | --- | --- |
| **№ of studies** | **Study design** | **Risk of bias** | **Inconsistency** | **Indirectness** | **Imprecision** | **Other considerations** | № of patients | | **Effect** | | **Certainty** |
|  |  |  |  |  |  |  | **Intervention** | **Control** | **Relative**  **(95% CI)** | **Absolute**  **(95% CI)** |  |
| **Excessive gestational weight gain (follow-up: 18 weeks; assessed with: Institute of Medicine (IOM) recommendations)** | | | | | | | | | | | |
| 1 | randomised trials | very serious^a,b^ | not serious | serious^c^ | serious^d^ | none | 75/121 (62.0%) | 102/139 (73.4%) | **OR 0.56** (0.32 to 0.98) | **127 fewer per 1,000** (from 265 fewer to 4 fewer) | ⨁◯◯◯ Very low |
| **Adequate gestational weight gain (follow-up: 18 weeks; assessed with: Institute of Medicine (IOM) recommendations)** | | | | | | | | | | | |
| 1 | randomised trials | very serious^a,b^ | not serious | serious^c^ | serious^e^ | none | 29/121 (24.0%) | 25/139 (18.0%) | **OR 1.40** (0.74 to 2.64) | **55 more per 1,000** (from 40 fewer to 187 more) | ⨁◯◯◯ Very low |
| **Insufficient gestational weight gain (follow-up: 18 weeks; assessed with: Institute of Medicine (IOM) recommendations)** | | | | | | | | | | | |
| 1 | randomised trials | very serious^a,b^ | not serious | serious^c^ | serious^e^ | none | 17/121 (14.0%) | 12/139 (8.6%) | **OR 1.92** (0.85 to 4.34) | **67 more per 1,000** (from 12 fewer to 204 more) | ⨁◯◯◯ Very low |
| **Gestational hypertension (follow-up: 18 weeks; assessed with: blood pressure >140/90 mmHg or use of antihypertensive medication after the first prenatal consultation)** | | | | | | | | | | | |
| 1 | randomised trials | serious^a^ | not serious | serious^c^ | serious^e^ | none | 21/124 (16.9%) | 33/143 (23.1%) | **OR 0.72** (0.36 to 1.45) | **53 fewer per 1,000** (from 133 fewer to 72 more) | ⨁◯◯◯ Very low |
| **Gestational diabetes mellitus (follow-up: 18 weeks; assessed with: WHO criteria, with information from medical records)** | | | | | | | | | | | |
| 1 | randomised trials | serious^f^ | not serious | serious^c^ | serious^e^ | none | 14/36 (38.9%) | 14/35 (40.0%) | **OR 0.95** (0.36 to 2.49) | **12 fewer per 1,000** (from 206 fewer to 224 more) | ⨁◯◯◯ Very low |
| **Preterm birth (follow-up: 18 weeks; assessed with: information from medical records)** | | | | | | | | | | | |
| 1 | randomised trials | serious^f^ | not serious | serious^c^ | serious^e^ | none | 3/91  (3.3%) | 3/108 (2.8%) | **OR 1.38** (0.25 to 7.49) | **10 more per 1,000** (from 21 fewer to 148 more) | ⨁◯◯◯ Very low |
| **Caesarean delivery (follow-up: 18 weeks; assessed with: information from medical records)** | | | | | | | | | | | |
| 1 | randomised trials | serious^f^ | not serious | serious^c^ | serious^e^ | none | 46/97 (47.4%) | 43/110 (39.1%) | **OR 1.35** (0.76 to 2.39) | **73 more per 1,000** (from 63 fewer to 214 more) | ⨁◯◯◯ Very low |
| **Preeclampsia (follow-up: 18 weeks; assessed with: information from medical records)** | | | | | | | | | | | |
| 1 | randomised trials | serious^f^ | not serious | serious^c^ | serious^e^ | none | 1/24  (4.2%) | 4/28 (14.3%) | **OR 0.33** (0.03 to 4.04) | **91 fewer per 1,000** (from 138 fewer to 260 more) | ⨁◯◯◯ Very low |

**CI: confidence interval; OR: odds ratio**

**Explanations**

a. Dropouts exceeding 20% in the number of participants and/or unbalanced losses between groups.

b. Differences between the outcome planned in the protocol and that reported in the study.

c. The intervention, in addition to the consumption of UPFs, included other dietary and physical activity recommendations.

d. Statistical significance changes depending on the use of conventional or modified intention-to-treat analysis.

e. The 95% confidence interval is consistent with the possibility of no effect or significant risk/benefit.

f. Information from medical records, not available for more than 20% of the participants.

**Supplementary Table S12:** GRADE assessment (Studies in women with grade I-II obesity)

**Bibliography:** Giacomello L, Bordignon S, Salm D, Donatello N, Belmonte LA, Bobinski F, Tourinho Dos Santos CF, Traebert JL, Piovezan AP, Martins DF. Effects of the application of a food processing-based classification system in obese women: A randomized controlled pilot study. Nutr Health (2023)2601060231153947. doi: 10.1177/02601060231153947

| **Certainty assessment** | | | | | | | | Summary of findings | |
| --- | --- | --- | --- | --- | --- | --- | --- | --- | --- |
| **№ of studies** | **Study design** | **Risk of bias** | **Inconsistency** | **Indirectness** | **Imprecision** | **Other considerations** | **Impact** | | Certainty |
| **Systolic blood pressure (follow-up: 12 weeks)** | | | | | | | | | |
| 1 | randomised trials | very serious^a,b,c^ | not serious | serious^d^ | not serious | none | There were no observed changes in the intervention group that received recommendations to avoid UPFs, nor in the control group that did not receive any intervention. The authors did not compare the differences between the groups at the end of the follow-up period. | | ⨁◯◯◯ Very low |
| **Diastolic blood pressure (follow-up: 12 weeks)** | | | | | | | | | |
| 1 | randomised trials | very serious^a,b,c^ | not serious | serious^d^ | not serious | none | There were no observed changes in the intervention group that received recommendations to avoid UPFs, nor in the control group that did not receive any intervention. The authors did not compare the differences between the groups at the end of the follow-up period. | | ⨁◯◯◯ Very low |
| **HDL cholesterol (follow-up: 12 weeks)** | | | | | | | | | |
| 1 | randomised trials | serious^b,c^ | not serious | serious^d^ | not serious | none | There were no observed changes in the intervention group that received recommendations to avoid UPFs, nor in the control group that did not receive any intervention. The authors did not compare the differences between the groups at the end of the follow-up period. | | ⨁⨁◯◯ Low |
| **Triglycerides (follow-up: 12 weeks)** | | | | | | | | | |
| 1 | randomised trials | serious^b,c^ | not serious | serious^d^ | not serious | none | There were no observed changes in the intervention group that received recommendations to avoid UPFs, nor in the control group that did not receive any intervention. The authors did not compare the differences between the groups at the end of the follow-up period. | | ⨁⨁◯◯ Low |
| **Body weight (follow-up: 12 weeks)** | | | | | | | | | |
| 1 | randomised trials | serious^b,c^ | not serious | serious^d^ | not serious | none | A significant reduction was observed in the intervention group that received recommendations to avoid UPFs (p<0.05). No changes were observed in the control group that did not receive any intervention. The authors did not compare the differences between the groups at the end of the follow-up period. | | ⨁⨁◯◯ Low |
| **Hip circumference (follow-up: 12 weeks)** | | | | | | | | | |
| 1 | randomised trials | serious^b,c^ | not serious | serious^d^ | not serious | none | A significant reduction was observed in the intervention group that received recommendations to avoid UPFs (p<0.05). No changes were observed in the control group that did not receive any intervention. The authors did not compare the differences between the groups at the end of the follow-up period. | | ⨁⨁◯◯ Low |
| **Waist circumference (follow-up: 12 weeks)** | | | | | | | | | |
| 1 | randomised trials | serious^b,c^ | not serious | serious^d^ | not serious | none | There was a significant reduction observed both in the intervention group that received recommendations to avoid UPFs (p<0.001), and in the control group that did not receive any intervention (p<0.05). The authors did not compare the differences between the groups at the end of the follow-up period. | | ⨁⨁◯◯ Low |
| **Quality of life - Domain: pain (follow-up: 12 weeks; assessed with: SF-36 questionnaire)** | | | | | | | | | |
| 1 | randomised trials | very serious^b,c,e^ | not serious | serious^c^ | not serious | none | There were no significant changes observed in the intervention group that received recommendations to avoid UPFs, nor in the control group that did not receive any intervention. The authors did not compare the differences between the groups at the end of the follow-up period. | | ⨁◯◯◯ Very low |
| **Quality of life - Domain: physical aspects (follow-up: 12 weeks; assessed with: SF-36 questionnaire)** | | | | | | | | | |
| 1 | randomised trials | very serious^b,c,e^ | not serious | serious^d^ | not serious | none | There were no significant changes observed in the intervention group that received recommendations to avoid UPFs, nor in the control group that did not receive any intervention. The authors did not compare the differences between the groups at the end of the follow-up period. | | ⨁◯◯◯ Very low |
| **Quality of life - Domain: general health status (follow-up: 12 weeks; assessed with: SF-36 questionnaire)** | | | | | | | | | |
| 1 | randomised trials | very serious^b,c,e^ | not serious | serious^d^ | not serious | none | There were no significant changes observed in the intervention group that received recommendations to avoid UPFs, nor in the control group that did not receive any intervention. The authors did not compare the differences between the groups at the end of the follow-up period. | | ⨁◯◯◯ Very low |
| **Quality of life - Domain: vitality (follow-up: 12 weeks; assessed with: SF-36 questionnaire)** | | | | | | | | | |
| 1 | randomised trials | very serious^b,c,e^ | not serious | serious^d^ | not serious | none | A significant improvement was observed in both the intervention group that received recommendations to avoid UPFs, and in the control group that did not receive any intervention (p<0.05). The authors did not compare the differences between the groups at the end of the follow-up period. | | ⨁◯◯◯ Very low |
| **Quality of life - Domain: mental health (follow-up: 12 weeks; assessed with: SF-36 questionnaire)** | | | | | | | | | |
| 1 | randomised trials | very serious^b,c,e^ | not serious | serious^d^ | not serious | none | A significant improvement was observed in both the intervention group that received recommendations to avoid UPFs, and in the control group that did not receive any intervention (p<0.05). The authors did not compare the differences between the groups at the end of the follow-up period. | | ⨁◯◯◯ Very low |
| **Quality of life - Domain: functional capacity (follow-up: 12 weeks; assessed with: SF-36 questionnaire)** | | | | | | | | | |
| 1 | randomised trials | very serious^b,c,e^ | not serious | serious^d^ | not serious | none | A significant improvement was observed in the intervention group that received recommendations to avoid UPFs (p<0.05), but not in the control group that did not receive any intervention. The authors did not compare the differences between the groups at the end of the follow-up period. | | ⨁◯◯◯ Very low |
| **Quality of life - Domain: social aspects (follow-up: 12 weeks; assessed with: SF-36 questionnaire)** | | | | | | | | | |
| 1 | randomised trials | very serious^b,c,e^ | not serious | serious^d^ | not serious | none | A significant improvement was observed in the intervention group that received recommendations to avoid UPFs (p<0.01), but not in the control group that did not receive any intervention. The authors did not compare the differences between the groups at the end of the follow-up period. | | ⨁◯◯◯ Very low |
| **Quality of life - Domain: emotional aspects (follow-up: 12 weeks; assessed with: SF-36 questionnaire)** | | | | | | | | | |
| 1 | randomised trials | very serious^b,c,e^ | not serious | serious^d^ | not serious | none | A significant improvement was observed in the intervention group that received recommendations to avoid UPFs (p<0.01), but not in the control group that did not receive any intervention. The authors did not compare the differences between the groups at the end of the follow-up period. | | ⨁◯◯◯ Very low |

**CI:** confidence interval; **OR:** odds ratio

#### Explanations

a. Differences in baseline characteristics between the intervention and control groups could lead to bias in estimating the effect of the intervention.

b. Pilot study. Dropouts of 30% were recorded in the intervention group and 50% in the control group.

c. Analyses of between-group differences at the end of the study are not available.

d. The intervention, in addition to the consumption of UPFs, included other dietary recommendations.

e. Open label trial. Knowledge of the intervention received could influence the participant-reported outcomes.

**Supplementary Table S13:** GRADE assessment (Studies in women with grade I-II obesity)

**Bibliography:** Chen F, Huang K, Long Q, Ma M, Zhang T, Dong G, Wu W, Ni Y, Hui C-C, Fu J. Comparative dietary effectiveness of a modified government-recommended diet with avoidance of ultra-processed foods on weight and metabolic management in children and adolescents: An open-label, randomized study. Asia Pac J Clin Nutr (2022) 31:282–293. doi: 10.6133/apjcn.202206_31(2).0014

| **Certainty assessment** | | | | | | | **Summary of findings** | |
| --- | --- | --- | --- | --- | --- | --- | --- | --- |
| **№ of studies** | **Study design** | **Risk of bias** | **Inconsistency** | **Indirectness** | **Imprecision** | **Other considerations** | **Impact** | **Certainty** |
| **Body mass index (follow-up: 12 weeks)** | | | | | | | | |
| 1 | randomised trials | serious^a^ | not serious | not serious | not serious | none | No significant differences were observed between a group that received recommendations for intensive reduction of UPFs (<1 serving per week) without calorie restriction, compared to a less intensive reduction of UPFs with calorie restriction. | ⨁⨁⨁◯ Moderate |
| **Fasting glucose (follow-up: 12 weeks)** | | | | | | | | |
| 1 | randomised trials | serious^a^ | not serious | not serious | not serious | none | No significant differences were observed between a group that received recommendations for intensive reduction of UPFs (<1 serving per week) without calorie restriction, compared to a less intensive reduction of UPFs with calorie restriction. | ⨁⨁⨁◯ Moderate |
| **Fasting insulin (follow-up: 12 weeks)** | | | | | | | | |
| 1 | randomised trials | serious^a^ | not serious | not serious | not serious | none | No significant differences were observed between a group that received recommendations for intensive reduction of UPFs (<1 serving per week) without calorie restriction, compared to a less intensive reduction of UPFs with calorie restriction. | ⨁⨁⨁◯ Moderate |
| **Total cholesterol (follow-up: 12 weeks)** | | | | | | | | |
| 1 | randomised trials | serious^a^ | not serious | not serious | not serious | none | No significant differences were observed between a group that received recommendations for intensive reduction of UPFs (<1 serving per week) without calorie restriction, compared to a less intensive reduction of UPFs with calorie restriction. | ⨁⨁⨁◯ Moderate |
| **Triglycerides (follow-up: 12 weeks)** | | | | | | | | |
| 1 | randomised trials | serious^a^ | not serious | not serious | not serious | none | No significant differences were observed between a group that received recommendations for intensive reduction of UPFs (<1 serving per week) without calorie restriction, compared to a less intensive reduction of UPFs with calorie restriction. | ⨁⨁⨁◯ Moderate |
| **Serum uric acid (follow-up: 12 weeks)** | | | | | | | | |
| 1 | randomised trials | serious^a^ | not serious | not serious | not serious | none | No significant differences were observed between a group that received recommendations for intensive reduction of UPFs (<1 serving per week) without calorie restriction, compared to a less intensive reduction of UPFs with calorie restriction. | ⨁⨁⨁◯ Moderate |
| **Fat mass percentage (follow-up: 12 weeks; assessed with: dual X-ray absorptiometry)** | | | | | | | | |
| 1 | randomised trials | serious^b^ | not serious | not serious | not serious | none | No significant differences were observed between a group that received recommendations for intensive reduction of UPFs (<1 serving per week) without calorie restriction, compared to a less intensive reduction of UPFs with calorie restriction. | ⨁⨁⨁◯ Moderate |

**CI:** confidence interval; **MD:** mean difference; **OR:** odds ratio

#### Explanations

a. Unbalanced dropouts in the control group could have influenced the true value of the outcome.

b. 43% of participants in the intervention group and 63% in the control group refused to be evaluated, which may lead to bias in the intervention effect estimate.

**Supplementary Table S14:** GRADE assessment (Studies in weight-stable adults)

**Bibliography:** Hall KD, Ayuketah A, Brychta R, Cai H, Cassimatis T, Chen KY, Chung ST, Costa E, Courville A, Darcey V, et al. Ultra-Processed Diets Cause Excess Calorie Intake and Weight Gain: An Inpatient Randomized Controlled Trial of Ad Libitum Food Intake. Cell Metab (2020) 32:690. doi: 10.1016/j.cmet.2020.08.014

| **Certainty assessment** | | | | | | | | **Summary of findings** | | | | |
| --- | --- | --- | --- | --- | --- | --- | --- | --- | --- | --- | --- | --- |
| **№ of studies** | **Study design** | **Risk of bias** | **Inconsistency** | **Indirectness** | **Imprecision** | **Other considerations** | № of patients | | | Effect | | **Certainty** |
|  |  |  |  |  |  |  | **Intervention** | | Control | **Relative**  **(95% CI)** | Absolute  (95% CI) |  |
| **Energy intake (follow-up: 4 weeks)** | | | | | | | | | | | | |
| 1 | randomised trials | very serious^a,b,c^ | not serious | not serious | not serious | none | 20 | | 20 | - | MD 508 Kcal/day higher (CI not provided) | ⨁⨁◯◯ Low |
| **Carbohydrate intake (follow-up: 4 weeks)** | | | | | | | | | | | | |
| 1 | randomised trials | very serious^a,b,c^ | not serious | not serious | not serious | none | 20 | | 20 | - | MD 280 Kcal/day higher (CI not provided) | ⨁⨁◯◯ Low |
| **Fat intake (follow-up: 4 weeks)** | | | | | | | | | | | | |
| 1 | randomised trials | very serious^a,b,c^ | not serious | not serious | not serious | none | 20 | | 20 | - | MD 230 Kcal/day higher (CI not provided) | ⨁⨁◯◯ Low |
| **Protein intake (follow-up: 4 weeks)** | | | | | | | | | | | | |
| 1 | randomised trials | very serious^a,b,c^ | not serious | not serious | not serious | none | 124 | | 143 | - | MD 2 Kcal/day lower (CI not provided) | ⨁⨁◯◯ Low |
| **Body weight (follow-up: 4 weeks)** | | | | | | | | | | | | |
| 1 | randomised trials | serious^a,c^ | not serious | not serious | not serious | none | Participants gaining 0.9 ± 0.3 kg (p =0.009) during the ultra-processed diet and losing 0.9 ± 0.3 kg (p = 0.007) during the unprocessed diet. | | | | | ⨁⨁⨁◯ Moderate |
| **Glucose (follow-up: 4 weeks)** | | | | | | | | | | | | |
| 1 | randomised trials | serious^a,c^ | not serious | not serious | not serious | none | No significant differences were observed between the values reached at the end of the ultra-processed diet period (88.6 ± 0.9 mg/dL) or the unprocessed diet period (88.0 ± 0.9 mg/dL) (p=0.62). | | | | | ⨁⨁⨁◯ Moderate |
| **Insulin (follow-up: 4 weeks)** | | | | | | | | | | | | |
| 1 | randomised trials | serious^a,c^ | not serious | not serious | not serious | none | No significant differences were observed between the values reached at the end of the ultra-processed diet period (11.3 ± 1.0) or the unprocessed diet period (8.9 ± 1.0) (p=0.09). | | | | | ⨁⨁⨁◯ Moderate |
| **Glycated hemoglobin (follow-up: 4 weeks)** | | | | | | | | | | | | |
| 1 | randomised trials | serious^a,c^ | not serious | not serious | not serious | none | No significant differences were observed between the values reached at the end of the ultra-processed diet period (5.02% ± 0.03) or the unprocessed diet period (5.00% ± 0.03) (p=0.64). | | | | | ⨁⨁⨁◯ Moderate |
| **Total cholesterol (follow-up: 4 weeks)** | | | | | | | | | | | | |
| 1 | randomised trials | serious^a,c^ | not serious | not serious | not serious | none | A significant reduction was observed at the end of the unprocessed diet period compared to the ultra-processed diet period (152 ± 3 mg/dL vs. 137 ± 3 mg/dL; p=0.001) | | | | | ⨁⨁⨁◯ Moderate |
| **HDL cholesterol (follow-up: 4 weeks)** | | | | | | | | | | | | |
| 1 | randomised trials | serious^a,c^ | not serious | not serious | not serious | none | A significant reduction was observed in both groups compared to baseline values. The reduction was greater at the end of the unprocessed diet period compared to the ultra-processed diet period (55.0 ± 0.9 mg/dL vs. 48.3 ± 0.8 mg/dL; p<0.0001) | | | | | ⨁⨁⨁◯ Moderate |
| **LDL cholesterol (follow-up: 4 weeks)** | | | | | | | | | | | | |
| 1 | randomised trials | serious^a,c^ | not serious | not serious | not serious | none | No significant differences were observed between the values reached at the end of the ultra-processed diet period (84 ± 3 mg/dL) or the unprocessed diet period (77 ± 3 mg/dL) (p=0.085). | | | | | ⨁⨁⨁◯ Moderate |
| **Triglycerides (follow-up: 4 weeks)** | | | | | | | | | | | | |
| 1 | randomised trials | serious^a,c^ | not serious | not serious | not serious | none | No significant differences were observed between the values reached at the end of the ultra-processed diet period (62 ± 3 mg/dL) or the unprocessed diet period (59 ± 3 mg/dL) (p=0.45). | | | | | ⨁⨁⨁◯ Moderate |

**CI:** confidence interval; **MD:** mean difference; **OR:** odds ratio

**Explanations**

a. The lack of blinding of participants could influence food consumption within each dietary period.

b. The lack of blinding of the assessors could influence the assessment of the outcomes.

c. A washout period was not employed to ensure that there is no carryover effect.
